# Risks and benefits of pasture irrigation using treated municipal effluent : a lysimeter case study, Canterbury, New Zealand

**DOI:** 10.1007/s11356-020-07759-8

**Published:** 2020-01-23

**Authors:** Maria Jesus Gutierrez-Gines, Minakshi Mishra, Cameron McIntyre, Henry Wai Chau, Juergen Esperschuetz, Roger McLenaghen, Mike P. Bourke, Brett H. Robinson

**Affiliations:** 1 Department of Soil and Physical Sciences, Lincoln University, Ellesmere Junction Road/Springs Road, Lincoln, 7647 New Zealand; 2 Institute of Environmental Science and Research Ltd, 27 Creyke Rd, Christchurch, 8041 New Zealand; 3 Christchurch City Council, 53 Hereford Street, Christchurch, 8154 New Zealand; 4 School of Physical and Chemical Sciences, University of Canterbury, 20 Kirkwood Ave, Christchurch, 8041 New Zealand

**Keywords:** Treated municipal effluent reuse, Soil sodicity, Nitrogen leaching, Soil phosphorus, Wastewater

## Abstract

Compared to discharge into waterways, land application of treated municipal effluent (TME) can reduce the need for both inorganic fertilizers and irrigation. However, TME irrigation may result in the accumulation of phosphorus (P) or trace elements in soil, and increased salinity and sodicity, which could damage soil structure and reduce infiltration. TME irrigation can also result in groundwater contamination through nitrate leaching or surface water contamination through runoff. This study aimed to evaluate the effects of increasing TME irrigation rates on quantity and quality of leachate and pasture growth in a lysimeter experiment using a Fluvial Recent soil and a Fragic Pallic soil. Pasture growth in the lysimeters was up to 2.5-fold higher in the TME treatments compared to the non-irrigated treatments. There were no signs of toxicity or accumulation of B, Al, Cd, Cu, Fe, Mn, As, and Zn. TME significantly increased the concentration of P and Na in the pasture. Nitrogen leaching from the lysimeters was negligible (< 1 kg/ha^−1^ equiv.) in all treatments, but mineral N accumulated in the soil profile of the highest application rate (1672 mm/yr). Although more P was added than removed in pasture, the rate of accumulation indicated that over a 50-year period, P will still be within the current New Zealand thresholds for grazed pastures. Sodium accumulated in the soil columns in all the TME treatments. The rate of accumulation was not proportional to the TME application rate, indicating that Na was moving down through the soil profile and leaching. Results indicate a low to moderate risk of sodicity in soil or toxicity in plants caused by Na.

**Electronic supplementary material:**

The online version of this article (10.1007/s11356-020-07759-8) contains supplementary material, which is available to authorized users.

## Introduction

Treated municipal effluent (TME) is a resource of water and plant nutrients, especially N and P, and lower concentrations of K, Zn, B and S (Pedrero et al. 2010). As well as alleviating drought stress, irrigation with TME offsets the need for mineral fertilizers such as superphosphate, which, depending on their origin, may contain elevated concentrations of toxic cadmium, fluorine and uranium (Kim and Robinson 2015).

There are environmental benefits for using TME for irrigation if the alternative is discharge into waterways or the ocean, where the nutrients that TME contains can exacerbate eutrophication and/or toxic algal blooms (Sonune and Ghate 2004). Apart from taking up nutrients, plant roots can mitigate pathogens (Prosser et al. 2016) and break down or immobilize contaminants (Chaudhry et al. 2005) that would otherwise degrade water bodies. Protecting freshwater and reusing the resources of TME are major drivers for irrigation with TME (pasture, crops, forestry, urban gardens, among others), which can make up > 20% of the irrigation water in water-scarce regions around the world (Pedrero et al. 2010).

Irrigation with TME carries risks that need to be assessed and mitigated for a successful operation (Cameron et al. 1997). Increased salinity and/or sodicity in the soils (Pedrero et al. 2010; Qian and Mecham 2005; Zalacáin et al. 2019) can reduce soil fertility (Abrol et al. 1988) and damage soil structure through the dispersion of clays (Mojid and Wyseure 2013), thereby reducing the permeability of soils (Tanji 1997). TME may add trace elements to soils (Xu et al. 2010), which could enter the food chain (Asgari and Cornelis 2015; Pedrero et al. 2010).

Although the effect on groundwater is usually mentioned as a potential risk (Lal et al. 2015; Rattan et al. 2005), only Barton et al. (2005) and Sparling et al.( 2006) directly analysed N and P in the leachates resulting from soils irrigated with wastewater. They reported that the N and P leached was 3–5% and < 1% in pumice and allophanic soils, respectively, of the total N and P applied by TME irrigation. This percentage increased to 22% for N and 8–13% for P in gley and recent soils. These are two of the main concerns about water quality in New Zealand (MfE and MPI 2018).

The balance between the benefits of the water and nutrients supplied by TME irrigation, and the risks posed by potential contaminants or excess of certain elements, is dependent on the quality of the TME, as well as environmental conditions, including soil type, vegetation cover and climate (Pedrero et al. 2010). Although there is much research on the potential risks and benefits of irrigation with TME, there is a scarcity of experimental evidence that assess comprehensively the positive and negative effects of TME irrigation in all the affected compartments in the system: plant cover, soil and leachates.

To address this lack of experimental data, above all in quantity and quality of leaching, a lysimeter experiment was set up for assessing the benefits and risks of increasing irrigation rates of TME over two soil profiles with distinct pasture types. The objectives of this experiment were:

- To measure the pasture production and its quality in two soils types with TME irrigation
- To determine whether TME irrigation would cause excessive leaching of N, P, Na or trace elements
- To assess whether there would be an unacceptable accumulation of P, Na or other elements in the soil

## Materials and methods

### Field sites and soils and treated municipal effluent

The two sites chosen for this experiment are currently under consideration for receiving TME from a wastewater treatment plant located in their proximity: a golf course and a grazed pasture. The Akaroa Golf Course is located in Duvauchelle, NZ (43°44′53.06”S, 172°55′41.44″E) over a Fluvial Recent soil (Barry’s soil, silt loam) (LandcareResearch 2018a), henceforth called Fluvial Recent soil site. The grazed pasture is located in Takamatua Peninsula (43°47′33.11”S, 172°57′16.96″E) over Fragic Pallic soil (Pawson silt loam) (LandcareResearch 2018a), referred to along the text as Fragic Pallic soil site. Before collecting the lysimeters, soil pits were opened to ascertain that they would have an adequate permeability to allow significant throughflow of water. Both soils are imperfectly drained (as indicated by mottling); however, there was no evidence of perched water or a fragipan. The particle size fractions for these soils were course sand 1.2 (s.d. 0.2) %, fine sand 44.5 (s.d. 0.9) %, silt 28.1 (s.d. 2.1) % and clay 24.0 (s.d. 2.2) % (Anon, 1939).

The TME was sourced from Duvauchelle wastewater treatment plant (43°45′07.16”S, 172°56′22.81″E). The wastewater received primary and secondary treatment within the plant followed by a UV disinfection. Table 1 shows the chemical characteristics of soils and TME.

**Table 1:** Characteristics of the treated municipal effluent (TME) used in the lysimeter experiment and the soil (A horizon) in the lysimeters

Parameters	TME	TME guidelines	Fluvial Recent soil	Fragic Pallic soil
pH	7.5	6.5–8.4 ^1^	4.8	5.2
EC (μS/cm)	423 (40)	700 ^1^	–	
Total suspended solids	32	450 ^1^	–	–
NH_4_^+^-N	0.49 (0.15–0.80)*		10.1 (7.5)	11 (6.8)
NO_3_^−^ -N	18 (7.5)	5 ^1^ – 10 ^2^	4.4 (1.1)	17.1 (13.2)
NO_2_^–^N	0.86 (0.09)		–	–
Total C (%)	–		4.4 (0.6)	5.4 (0.3)
Total N (%)	< 0.025		0.38 (0.05)	0.48 (0.03)
Al	0.43 (0.11–1.7)*	5 ^2^	34,900 (3700)	32,700 (1420)
B	0.10 (0.04)	0.7 ^1^		–
Ca	59 (12)		5850(187)	6770 (393)
Cd	< 0.001	0.01 ^2^	< 0.05	<0.05
Cu	0.04 (0.03)	0.2 ^2^	5.1 (1.4)	7.7 (0.2)
Fe	0.96 (0.25–3.6)*	5 ^2^	16,800 (4100)	20,200 (2850)
K	22 (5.0)		4008 (365)	4490 (346)
Mg	19 (5.5)		3580 (463)	4250 (76)
Mn	0.06 (0.03)	0.2 ^2^	496 (50)	624 (9)
Na	95 (21)	69 ^1^	374 (30)	290 (10)
P	11 (5.0)	5 ^2^	599 (125)	1050 (30)
S	25 (11)		430 (5)	490 (21)
Zn	0.17 (0.11)	2 ^2^	62 (7)	68 (3)
SAR	2.75		–	

*Geometric mean and standard deviation range

^1^FAO (Food and Agriculture Organization) guidelines for unrestricted reuse (FAO 2003)

^2^EPA (US Environmental Protection Agency) guidelines for agricultural reuse (EPA 2004)

Values in brackets represent the standard deviation of the mean. TME, *n* = 54 except trace elements *n* = 14. Concentrations of dissolved elements in TME are in mg/L, and the total elements in soils are expressed in mg/kg, unless otherwise indicated

### Lysimeter experiment set up and monitoring

Lysimeters comprised intact soil cores (80 cm deep and 50 cm diameter) collected following the method of Cameron et al. (1992): they were excavated around a cylinder to minimize soil disturbance, with a 5-cm layer of gravel to allow for leaching. Molten petroleum jelly was injected around the edge of the lysimeter to eliminate bypass flow. Prior to the setup of the full lysimeter experiment, two intact lysimeters were collected from the Fluvial Recent soil site to test that the intact soil cores drained and therefore were suitable for the full experiment. These two lysimeters were taken to Lincoln University lysimeter facility (43°38′53.54”S, 172°28′7.69″E) and irrigated with 2 L of water (10 mm) per day for 6 weeks until drainage was stabilized. After that, a further ten lysimeters were taken from the Fluvial Recent soil site and six were taken from the Fragic Pallic soil site. The original vegetation was kept in the lysimeters. The Fluvial Recent soil lysimeters were covered with a fescue/browntop (*Festuca* sp./*Agrostis capillaris* L.) mixture, which is a common golf course turf. These species grow densely but not very tall (compared with ryegrass), and they do not require intensive maintenance and fertilization. The Fragic Pallic soil lysimeters were dominated by perennial ryegrass (*Lolium perenne* L.), which is characterized by a high biomass production but needs maintenance and frequent fertilization compared with the golf course turf.

From 9 February to the 21 April 2015, the lysimeters were irrigated with 10 mm (2 L) of water per day, to provide drainage a reasonable timeframe (about 2 weeks) without an unrealistically high water input. After 10 days, all the lysimeters started to drain, and after 6 weeks, similar volumes of leachate were obtained for all lysimeters; this irrigation was kept until 22 April, when TME application of the lysimeters began. TME was collected by the Christchurch City Council (CCC) from the Duvauchelle wastewater treatment plant and delivered to Lincoln University in a 1000-L tank. Samples of the stored TME were collected and analysed weekly. The tank was refilled as needed. The chemical characteristics of the TME tank samples (Table 1) were similar to data provided by CCC from various times during the past 5 years (data not shown). Irrigation treatments were chosen to represent approximately half of the annual rainfall in the area (low TME application), same as the annual rainfall (medium TME application) and double the annual rainfall (high TME application). There were three replicates per treatment (Table 2).

**Table 2:** Experimental design with two soil types and up to four TME irrigation rates and three replicate lysimeters per treatment

Soil type / Irrigation	0 mm/yr	446 mm/yr	836 mm/yr	1672 mm/yr
Fluvial Recent soil	X 3	X 3	X 3	X 3
Fragic Pallic soil	X 3	–	X 3	–

TME was irrigated daily from the stored tank with a watering can for the period of 17.5 months (until 9 October 2016). The TME in the tank was homogenized every day before TME application with a hand stirrer. Drainage volumes were measured and collected weekly or more often following high rainfall events. Pasture was harvested periodically, typically every 3 weeks during the growing season or every 2 months over winter.

The experiment was conducted for 17.5 months, covering two wet seasons. At the end of the experiment, pasture was harvested for the final time, and the lysimeters were deconstructed. Soil samples were collected and analysed from 0–15, 15–30, 30–45 to 45–60 cm.

### Sample preparation and chemical analyses

Samples of both the TME storage tank (duplicates) and the leachates were collected weekly. One sample per week from the TME storage tank was filtered through a 0.45-μm syringe filter and frozen at − 20 °C until analysis. Other sample was frozen without filtering. All the leachate samples were filtered and stored at − 20 °C until analysis. NO_3_^−^-N and NH_4_^+^-N were measured weekly in tank samples and leachates using a flow injection analyser (FIA FS3000 twin channel analyser, Alpkem, USA). pH was determined with a pH meter and conductivity meter (Mettler Toledo Seven Easy) and total C and N with a Vario-Max CN Elementar Analyser (Elementar®, Germany). Every 2 months, acidified (using 1 mL of 6 M HNO_3_ into 30 mL) subsamples of leachates and TME were analysed for As, B, Ca, Cd, Cr, Cu, Fe, K, Mg, Mn, Mo, Na, P, S and Zn using inductively coupled plasma optical emission spectrometry (ICP-OES Varian 720 ES, USA). Unfiltered TME samples were also microwaved, digested (see method below) and analysed by ICP-OES.

The fresh samples of soils were sieved (< 2 mm), and NO_3_^−^-N, and NH_4_^+^-N concentrations were determined on 2-M KCl extracts (Clough et al. 2001) and analysed with a flow injection analyser (FIA FS3000 twin channel analyser, Alpkem, USA). Sieved samples of soil were then dried at room temperature and analysed for pH and electrical conductivity in a 1:5 (w:v) soil-water ratio (Blakemore et al. 1987). Total C and N concentrations were determined using a Vario-Max CN Elementar Analyser. Pseudo-total elements were extracted using the microwave CEM MARS Xpress acid digest technique (0.5-g substrate, 4.0-ml trace element grade nitric acid (69%) and 4.0 ml 30% hydrogen peroxide), according to the equipment specifications. The Olsen P was extracted with 0.5-M NaHCO_3_ (Olsen et al. 1954). Exchangeable cations were extracted with 0.01-M AgTU^+^ (silver thiourea) (Blakemore et al. 1987).

Samples of pasture were washed with tap water to remove soil and oven-dried at 65 °C until constant weight was obtained. Dried samples were weighed and ground and passed through a 0.5-mm stainless steel sieve.Total C and N concentrations were determined using a Vario-Max CN Elementar Analyser. Arsenic, Ca, Cd, Cu, K, Mg, Pb, S and Zn were analysed following acid digestion in a microwave (CEM MARS Xpress), using 0.3-g dried plant material ,2.0-ml trace element grade nitric acid (69%) and 2.0 ml 30% hydrogen peroxide, according to the equipment specifications.

Analysis of elements in the extracts of soil and plants was determined by ICP-OES and expressed on a dry weight basis. The microwave extraction method was assessed using a reference soil (reference 981, sandy soil from the Netherlands) and a reference plant sample (reference 952, mixture of grasses from the Netherlands) from Wageningen Evaluating Programs for Analytical Laboratories (WEPAL, NL-6700 EC Wageningen, the Netherlands). Recoverable concentrations of the reference materials were within 93–110% of the certified values. For soils and plants, the detection limit (LD) was 0.05 mg/kg for most elements, and the quantification limit (LQ) was 0.1 mg/kg. For solutions the LD and LQ were 50 times lower.

### Data analysis

The sodium adsorption ratio (SAR) in soils and TME were calculated based on Ayers and Westcot (1985), using exchangeable Ca, Mg and Na data in meq/L or meq/kg. Exchangeable sodium percentage (ESP) in soils was calculated using exchangeable Na, Ca and Mg data in cmol/kg (Abrol et al. 1988).

Data were analysed using Minitab® 17 (Minitab Inc., State College, Pennsylvania, USA) and Microsoft Excel 2013. The results from the two soils were analysed separately. For the results of the Fluvial Recent soil, the ANOVA with Tukey’s post hoc test was used to assess the effects of different treatments. For the results of the Fragic Pallic soil, the t-test not assuming equal variances was used to assess the both treatments. The significance level for all statistical analyses was *P* < 0.05.

### Calculations of phosphorus fluxes

Data of P in TME, soil, leachate and biomass were used to calculate the P accumulation in the topsoil (0–30 cm) in a period of 50 years with 500-mm TME applied per year and three P concentrations in the TME. Calculations were done for the 0–30-cm topsoil because it is a typical plough depth and is where the most root biomass is found. The parameters used in the model are shown in Table 3. The total concentration of P in the soil was calculated according to Eq. (1):

1 $$ Total\ P{\left( mg/ kg\right)}_n=P\  mass{\left( kg\ P/ ha\right)}_nx\ 1000/ Soil\ mass\left(t/ ha\right) $$

where *P mass* is the total amount of P in kg/ha in the top 30 cm of soil in a certain year (n); and *Soil mass* is calculated in the 0–30 cm horizon with 1.4 t/m^3^ density.

**Table 3:** Parameters used to simulate the risk of P accumulation in the topsoil with TME irrigation

Parameter	Fluvial Recent soil	Fragic Pallic soil
Effluent P concentration (mg/L)	5, 10 or 15	5, 10 or 15
Effluent application rate (mm/yr)	500	500
P application rate (kg/ha/yr)	25, 50, or 75	25, 50 or 75
Water flux (mm)^1^	482	400
Initial soil P concentration (mg/kg)	599	1046
Initial Olsen P (mg/kg)	11	41
Initial water-soluble P (CaCl_2_) (mg/L)^2^	0.048	0.18
Soil density (t/m^3^)	1.4	1.4
Simulation depth (m)	0.3	0.3
Biomass production (t/ha/yr)^3^	5.4	6.8

^1^Estimated from rainfall (969 mm/yr) + TME irrigation (500 mm/yr) – evapotranspiration (987 mm/yr for *Festuca* sp./ *Agrostis capillaris* L. and 1068 mm/yr for *Lolium perenne* L.)

^2^Estimated from ratios with Olsen P on similar soils from McDowell and Condron (2004) and Sánchez-Alcalá et al. (2014)

^3^Interpolated from data from lysimeters, for each type of plant cover, for total water irrigation of 1496 mm/yr

For calculating the changes of P mass in the soil, the simulations assumed that all the P that is applied in the TME, and is not taken up by plants, or leached to deeper horizons, will accumulate in the soil, as exposed in Eq. (2). Loss by run off was not considered.

2 $$ P\  mas s{\left( kg\ P/ ha\right)}_n=\left(P\  mas{s}_{n-1}+P\  application\right)\hbox{--} \left(P\  leache{d}_{n-1}+P\  uptak{e}_{n-1}\right) $$

The initial *P mass* (*P mass*_n0_) was calculated based on the initial P concentration in the soil (Tables 1 and 3). Application of P by 500 mm/yr of TME irrigation would depend on the P concentration in the TME, as shown in Table 3. *P leached* will depend on the water-soluble P, and the water flux, as shown in Eq. (3):

3 $$ P\  leached{\left( kg\ P/ ha/ yr\right)}_n= Water\ soluble\ P{\left( mg/L\right)}_n xWater\ flux(mm)/100 $$

*Water-soluble P* was calculated as a fraction of Olsen extractable P (McDowell and Condron 2004; Sánchez-Alcalá et al. 2014). Initial Olsen P and the fraction of Olsen P/Total P were those measured in the control lysimeters in the two soils. Fractions Olsen P/Total P and Soluble P/Olsen P were considered constant based on historical P unpublished data from Lincoln University and on McDowell and Condron (2004) and Sánchez-Alcalá et al. (2014).

The water flux was calculated as shown in Eq. (4):

4 $$ Water\ flux(mm)= Irrigation\left(500\  mm\right)+ Rainfall(mm)- Evapotranspiration(mm) $$

*Evapotranspiration* was calculated interpolating the lysimeter data for each type of plant cover (Table 4) for the assumed rainfall and irrigation values, and *Rainfall* is the average annual rainfall in Banks Peninsula, NZ, in the last 20 years (Macara 2016).

**Table 4:** General parameters for the length of the experiment (17.5 months)

Treatment	Total irrigation (mm)	Total drainage (mm)	Total evapotranspiration (mm)	Biomass production (t/ha equiv.)
*Fluvial Recent soil*				
Control	0	169 ± 22 ^a^	610	5.4 ± 1.0 ^a^
446 mm/yr	632	485 ± 23 ^b^	926	6.3 ± 0.6 ^ab^
836 mm/yr	1185	736 ± 17 ^c^	1228	8.9 ± 0.6 ^b^
1672 mm/yr	2370	1375 ± 11 ^d^	1774	12.3 ± 0.2 ^c^
*Fragic Pallic soil*				
Control	0	148 ± 2.0 ^a^	631	6.0 ± 0.3 ^a^
836 mm/yr	1185	609 ± 32 ^b^	1355	13.3 ± 0.7 ^b^

Total rainfall was 779 mm. Mean ± standard error (*n* = 3). For each soil type, values with the same letter are not significantly different (*P* < 0.05). The Fluvial Recent soil and Fragic Pallic soil were tested independently

Plant P uptake was calculated separately for each type of plant cover, based on lysimeter data. The pasture production was considered constant over the 50 years, since the results showed that water is the most limiting factor for plant growth. It was assumed that biomass was not limited by nutrients. Biomass production was interpolated from lysimeter data for each type of plant cover based on water supply of 1469 mm/yr (rain + TME irrigation).

## Results and discussion

### Water balance and biomass

Irrigation with TME increased the drainage of the lysimeters (Table 4). In the Fluvial Recent soils without TME application, drainage was 22% of the input water (rainfall only), while in the treatment with the medium irrigation rate (836 mm/yr), this percentage was 37%. In the case of Fragic Pallic soil, drainage was 19% of rainfall in control lysimeters, compared with 31% in the TME applied at 836 mm/yr. Although some authors reported a decreased infiltration rate in the long term (Bedbabis et al. 2014; Sparling et al. 2006), in the experiment, all the lysimeters receiving TME, even at the highest application rate, drained throughout the experiment. There was no ponding or visible evidence that the soil structure had been degraded.

Irrigation with TME significantly increased the biomass production in all the treatments (Table 4). The ryegrass growth in the Fragic Pallic soil lysimeters was significantly higher than the fescue/brown top mixture in the lysimeters containing Fluvial Recent soil: a 121% increase compared with 65% increase with the same TME irrigation rate. This is likely due to differences in species composition as well as previous soil management. The Fragic Pallic soil had higher fertility than the Recent Fluvial soil (Table 1). Increased biomass or yield is a general benefit of irrigation with TME. Such increases have been reported in studies with barley (Mohammad Rusan et al. 2007), olive trees (Bedbabis et al. 2015), pasture (Barton et al. 2005) and lettuce (Urbano et al. 2017) and other crops.

**Fig. 1:**
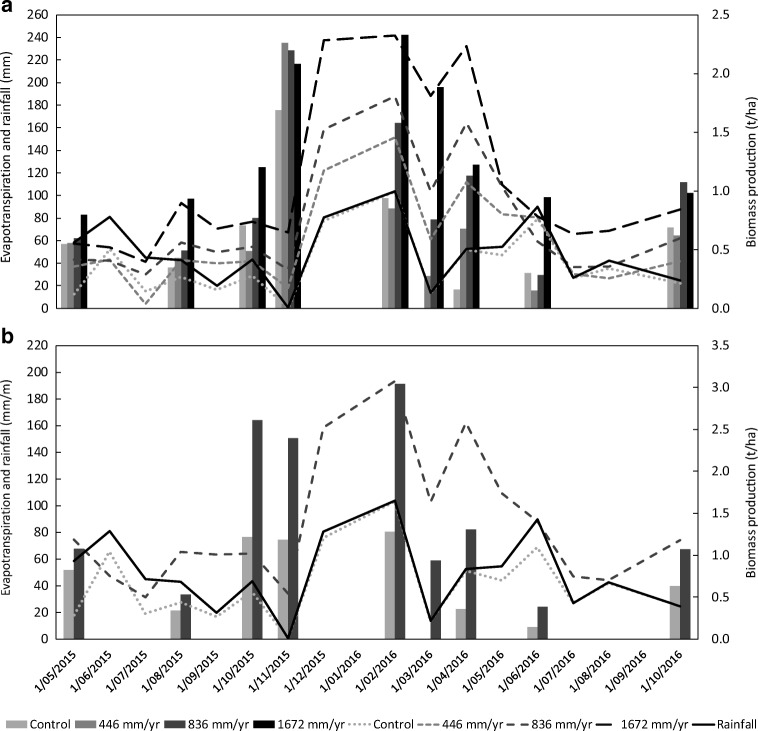
Evapotranspiration (rainfall + irrigation – leachate), rainfall and biomass production during the experiment. Bars represent the biomass produced in each sampling event. Lines represent the cumulative evapotranspiration and rainfall each month. **(A)** Results for Recent Fluvial soil lysimeters and **(B)** for Fragic Pallic soil lysimeters

Figure 1 shows the seasonality of the water balance, rainfall and pasture production. The biggest differences in evapotranspiration between treatments happened during the warmest months (December to April). The biggest difference in biomass production between treatments also happened during this period. This was remarkable in March and April 2016, when there was negligible plant growth in the control lysimeters (Fig. 1 and Supp. Material), indicating that irrigation was essential to maintain production. This is especially relevant for climate change scenarios, where the East Coast of New Zealand is forecasted to have drier summers, with rainfall less evenly distributed along the year (MfE 2018) and more need of alternative water sources.

Only 1% of the total drainage in lysimeters irrigated with 836 mm/yr TME occurred between October and April in both soil types (see Supp. Material). This percentage increased to 7% in the case of 1672 mm/yr application rate. This implies that, if possible, irrigation rates can be optimized in each season to maximize pasture production and minimize leaching.

### Nutrient balances

The average concentration of NO_3_^−^ in the TME (18 mg/L) was almost twice the maximum value (10 mg/L) for unrestricted irrigation of TME onto agricultural land according to guidelines in several states from the USA and other countries such as Arabia Saudi (EPA 2004) or for disposal in rivers according to the European Union (EEC 1991). The total P in the TME (11 mg/L) was fivefold higher than the maximum concentration allowed for TME disposal in surface water in the European Union (1–2 mg/L, EEC, 1991), or some states in the USA (1 mg/L, EPA, 2004), and twice the guideline value for unrestricted use on agricultural land (EPA, 2004).When discharged into water bodies, N and P can exacerbate algal blooms and reduce water quality (Leip et al. 2015). Reused in irrigation at the medium rate (836 mm/yr), TME would supply 146 kg N/ha/yr and 120 kg/P/ha/yr, as well as other nutrients such as S, K, Mg and Ca (Supp. Material), which, at the price of the lowest cost fertilizer in NZ, the total fertilizing value of this effluent at an application rate of 500 mm/yr is about US$ 840ha/yr (Table 5). Note that the total value of the nutrients is less than the sum of the individual elements because some fertilizers contain more than one element.

**Table 5:** Mass and value of plant macronutrients added through irrigating treated municipal effluent at a rate of 500 mm per year

Element	Mass (kg/ha/yr)	Value of element in cheapest fertilizer (US$/ha/yr)
N	95	72
P	55	135
K	110	201
S	125	262
Mg	95	175
Ca	295	249

The value was calculated from the cheapest fertilizer (Ballance 2017)

In contrast to the findings of other authors (Barton et al. 2005; Mohammad Rusan et al. 2007), TME irrigation did not affect pasture’s N concentration (Table 6), although it significantly increased the total amount of N taken up, due to the increased pasture growth. On the contrary, P concentration increased in proportion to the TME irrigation rates (Table 6), as also reported by previously cited authors. This indicates that N was, at least partially, a limiting nutrient for both types of pasture, while in the case of P, pasture presented a luxury uptake (McLaren and Cameron 1996). For TME irrigation rates up to 836 mm/yr, pasture extracted similar or more N than that supplied by TME. If the experiment had included a second growing season, a higher N uptake would have likely been demonstrated. Therefore, it would be the case that pasture could remove the N added with TME at rates above 1000 mm/yr. This is consistent with the findings of other studies investigating TME land application (Barton et al. 2005; Mohammad Rusan et al. 2007).

**Table 6:** Mass of N and P (kg/ha equiv., unless otherwise indicated) in the TME, pasture, soil and drainage water over the entire lysimeter experiment (17.5 months)

Treatment	Irrigation	Pasture concentration (*N* %, P mg/kg)	Pasture uptake	Soil total (0–60 cm) (N t/ha)	Soil mineral N/Olsen P (0–60 cm)	Leached
Nitrogen						
*Fluvial Recent soil*						
Control	0	2.14 ± 0.06 ^ab^	115 ± 21 ^a^	15.8 ± 2.0 ^a^	74 ± 12 ^a^	0.32 ± 0.03 ^a^
446 mm/yr	111	1.97 ± 0.08 ^b^	124 ± 14 ^ab^	13.4 ± 2.0 ^a^	63 ± 6 ^a^	0.72 ± 0.08 ^ab^
836 mm/yr	207	2.18 ± 0.01 ^ab^	193 ± 14 ^b^	13.1 ± 1.0 ^a^	95 ± 6 ^a^	1.09 ± 0.03 ^b^
1672 mm/yr	415	2.32 ± 0.05 ^a^	288 ± 113 ^c^	15.0 ± 1.3 ^a^	161 ± 17 ^b^	1.97 ± 0.18 ^c^
*Fragic Pallic soil*						
Control	0	2.52 ± 0.09 ^a^	151 ± 13 ^a^	19.7 ± 1.2 ^a^	78 ± 16 ^a^	0.37 ± 0.06 ^a^
836 mm/yr	207	2.36 ± 0.07 ^a^	314 ± 11 ^b^	19.0 ± 0.8 ^a^	91 ± 17 ^a^	1.05 ± 0.05 ^b^
Phosphorus						
*Fluvial Recent soil*						
Control	0	2277 ± 99 ^a^	13 ± 2 ^a^	4140 ± 464 ^a^	43 ± 6.3 ^a^	<1
446 mm/yr	77	2722 ± 91 ^ab^	16 ± 2 ^ab^	3500 ± 641 ^a^	29 ± 7.1 ^a^	<1
836 mm/yr	144	2960 ± 94 ^bc^	25 ± 3 ^b^	3410 ± 165 ^a^	29 ± 0.8 ^a^	<1
1672 mm/yr	289	3382 ± 113 ^c^	40 ± 1 ^c^	3720 ± 415 ^a^	33 ± 4.9 ^a^	<1
*Fragic Pallic soil*						
Control	0	3286 ± 160 ^a^	20 ± 2 ^a^	6020 ± 485 ^a^	164 ± 20 ^a^	<1
836 mm/yr	144	3502 ± 145 ^a^	45 ± 2 ^b^	5670 ± 100 ^a^	120 ± 4.2 ^a^	<1

Mean ± standard error (*n* = 3). For each soil type, values with the same letter are not significantly different *P* < 0.05)

In the highest irrigation rate (1672 mm/yr), the mass of N added was 1.4 times greater than that taken up by pasture. That was the only treatment that showed an accumulation of N in the soil, mainly as mineral nitrogen (NH_4_^+^, NO_3_^−^). In this study, the accumulation of inorganic N had only a small effect on the soil total N because total N > > inorganic N. However, other authors have reported significant increases in total N after 5–10 years of TME irrigation (Bedbabis et al. 2015; Mohammad Rusan et al. 2007). The total inorganic N leached from all treatments was < 2 kg/ha equiv. This was negligible compared to the > 40 kg/N/ha/yr that can be leached from a grazed pasture (Menneer et al. 2004). It was also lower than the NO_3_^−^ leached (4.5–22 kg N/ha/yr) reported by Barton et al. (2005), following TME irrigation at a rate of 400 kg/N/ha/yr.

**Fig. 2:**
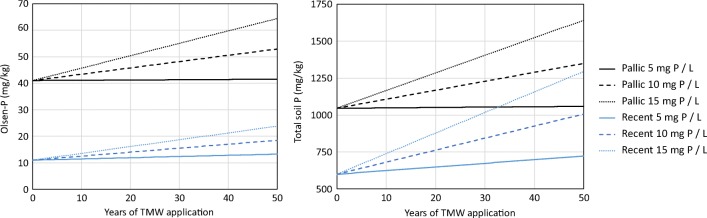
Calculated P in the top 30 cm of the Fragic Pallic soil and Fluvial Recent Soil under irrigation with TME at 500 mm/yr with a P concentration of 5, 10 or 15 mg/L. The parameters used for the calculations are given in Table 3, and the equations are 1 to 4

Even after P luxury uptake, TME irrigation added five to seven times more P than the removed by the pasture, and there was negligible P leaching (Table 6). The accumulation of P in the soil, or topsoil, was not detectable because the extra P applied (120 kg/ha) was 30-fold lower than the P concentration in soil. Over several years, P is likely to accumulate in the topsoil. Significant increases in P after 5 to 10 years of TME irrigation was reported by Barton et al. (2005), Mohammad Rusan et al. (2007) and Qian and Mecham (2005). The potential accumulation of P in the top soil calculated for a period of 50 years of TME irrigation is shown in Fig. 2. With a P concentration in the TME similar to the one of this experiment (10 mg/L), over a 50-year period of the total P concentration in the top 30 cm would increase from 1046 to 1349 mg/kg in the Fragic Pallic soil and from 599 to 1006 mg/kg in the Fluvial Recent soil (Fig. 2). Even with this increase, the total concentration at the end of the 50-year period would still be well within the range of P concentrations for NZ agricultural soils reported by McDowell and Condron (2004) and Reiser et al. (2014).

In the nominal case, the Olsen-extractable P in these soils is likely to increase from 41 to 53 mg/kg in the Fragic Pallic soil and increase from 11 to 18 mg/kg in the Fluvial Recent soil. The initial Olsen P concentration in the Fragic Pallic soil was within the range recommended by Dairy NZ (35–40 mg/kg) to maintain high productivity on sedimentary soils (DairyNZ 2012). This is undoubtedly a result of the soil management under the previous land use, grazed pasture. In contrast, the Fluvial Recent soil, with an initial Olsen P concentration of 11 mg/L is consistent with non-productive but managed land, in this case a golf course. Even with an increase to 18 mg/kg, the plant-available P would only be sufficient for low P-requiring crops such as golf course turf or for winter wheat (Tang et al. 2009). For pasture, Olsen *P* values above 100 mg/kg are excessive, and values are considered “high” from 50 to 100 mg/kg (LandcareResearch 2018b).

Phosphorous from a TME-irrigated area could enter waterways via runoff, particularly if it is an easily erodible area. In that case, it could cause serious environmental issues (Tilman et al. 2001). However, the loss of P from a cut-and-carry pasture irrigated by TME will always be lower than the losses from a grazed pasture (TME irrigated or otherwise) because of the mechanical disturbance of soil by the animals’ hooves (McDowell et al. 2003).

Although S, K, Ca and Mg are usually present at high concentrations in TME (Bedbabis et al. 2014; Qian and Mecham 2005), their behaviour in soil, plant or leachates is not as well studied as N and P, probably because they are not usually linked to excessive eutrophication (Garnier et al. 2010; Leip et al. 2015). In this experiment, concentrations of S, K, Ca and Mg in pasture did not increase in response to the TME treatments. Nevertheless, the total nutrients extracted by the pasture in the treatments was higher due to the increased biomass (Tables S1 to S4). Uptake of S, Ca and Mg was between 8 and 17% of the added amount in the 836-mm/yr treatments and 34 and 70% of the K added in Recent Fluvial and Fragic Pallic soils, respectively. Only small amounts of these nutrients leached (up to 13% in the 836-mm/yr treatments), so most will accumulate in the soil. The accumulation in soil was insignificant because the soil concentrations were at least 100-fold greater than the amount being added and a difference might be only noticeable in the long term (Bedbabis et al. 2014; Bedbabis et al. 2015; Mohammad Rusan et al. 2007; Qian and Mecham 2005). The addition of Mg and Ca will offset the Na supply, and will help maintaining the soil structure (Abrol et al. 1988). The application of any element to a system at a rate greater than the rate that is removed is ultimately unsustainable (Mills et al. 2005).

### Sodium and trace elements

**Fig. 3:**
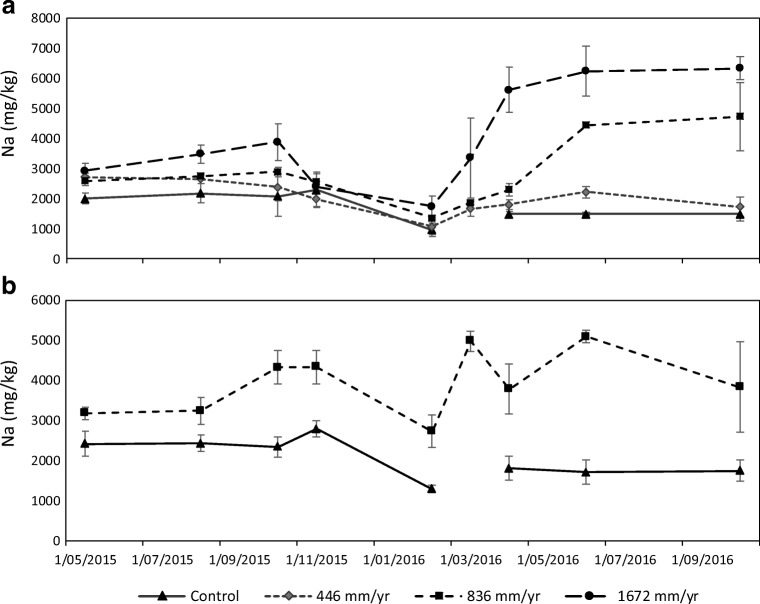
Sodium concentration in pasture (mg/kg) along the experiment. Bars represent standard error of the mean (*n* = 3). **a** Results from lysimeters of Fluvial Recent soil. **b** Results from lysimeters of Fragic Pallic soil. There was no biomass in control lysimeters in March 2016 (missing point)

The TME contained 95 mg/L Na, which is about 50% higher than the FAO guidelines for unrestricted use of effluent (FAO 2003). The SAR of the TME was 2.75, which when assessed in combination with EC (0.42 dS/m) indicates that the TME has a “slight to moderate” use restriction, in terms of maintaining the soil structure and crop production (Ayers and Westcot 1985). Table 7 shows that TME irrigation added more Na to soil than the amount taken up by the pasture. At the medium application rate (836 mm/yr), fescue/browntop and ryegrass uptake were 2 and 4%, respectively, of the applied Na. The Na concentration in pasture increased when increasing TME application rates (Table 7). The differences of plant Na between treatments were higher in the second half of the experiment (Fig. 3), above all for medium (836 mm/yr) and high (1672 mm/yr) rates. Zalacáin et al. (2019) also reported an increased Na concentration in leaves of plants irrigated with TME over a 15-year period. Elevated concentrations of Na in pasture increase its palatability to stock (Chiy et al. 1998), and farmers occasionally apply Na to their pastures for this reason. Although most pasture species are not overly sensitive to Na, the maximum concentration found in leaves (0.6%) can be toxic for some sensitive plants (Ayers and Westcot 1985).

**Table 7:** Mass of Na (kg/ha equiv) in the treated municipal effluent, pasture, soil and drainage water during the experiment

Treatment	Irrigation Na (kg/ha equiv.)	Average pasture Na (mg/kg)	Pasture Na (kg/ha equiv.)	Soil Na (0–60 cm) (kg/ha equiv.)	Na leached (kg/ha equiv.)
*Fluvial Recent soil*					
Control	0	1741 ± 270 ^a^	10 ± 3 ^a^	2598 ± 102 ^a^	45 ± 6 ^a^
446 mm/yr	605	2028 ± 205 ^a^	13 ± 3 ^a^	3031 ± 156 ^ab^	159 ± 18 ^b^
836 mm/yr	1131	2836 ± 138 ^ab^	23 ± 3 ^a^	3195 ± 149 ^ab^	264 ± 23 ^b^
1672 mm/yr	2256	4002 ± 499 ^b^	45 ± 6 ^b^	3349 ± 170 ^b^	412 ± 61 ^c^
*Fragic Pallic soil*					
Control	0	2121 ± 85 ^a^	13 ± 1 ^a^	2394 ± 54 ^a^	30 ± 0 ^a^
836 mm/yr	1131	3813 ± 348 ^b^	50 ± 2 ^b^	2919 ± 89 ^b^	232 ± 32 ^b^

Mean ± standard error. For each soil type, values with a different letter are significantly different (*P* < 0.05)

**Fig. 4:**
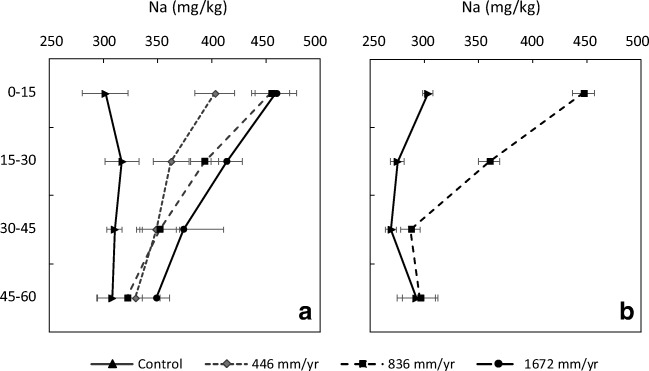
Soil Na concentration (mg/kg) as a function of depth at the end of lysimeter experiment for the Fluvial Recent soil **(a)** and Fragic Pallic soil **(b)**. Bars represent the standard error of the mean (*n* = 3)

About 20% of the Na applied by medium TME application rate (836 mm/yr) leached (Table 7). It also accumulated in the soil profile, mainly in the top horizons 0–15 cm and 15–30 cm (Fig. 4). This was also reported by Bedbabis et al. (2015), Qian and Mecham (2005) and Zalacáin et al. (2019). The control topsoil had ca. 300-mg/kg Na, whereas the topsoil under the low TME treatment (446 mm/yr) contained significantly higher concentrations (ca. 400 mg/kg). However, at the medium TME irrigation rate (836 mm/yr, i.e., double the “low” rate), Na only increased to ca. 450 mg/kg. Quadrupling the “low” TME irrigation rate increased the topsoil Na concentration to 460 mg/kg. The results indicate that at soil concentrations > ca. 400 mg/kg, most additional Na was not retained by the soil and leached down through the soil profile (Table 7 and Fig. 4). Similar findings were also shown by Bedbabis et al. (2014), who demonstrated that soil Na increased 4.3 times after 5 years of 500 mm/TME/yr application and was only five times higher (compared with initial soil concentration) after 10 years of irrigation. SAR and ESP values in the topsoil follow the same pattern (see Supp. Material), with an increase between control soil and the 446-mm/yr treatment (SAR from 0.87 to 1.48, ESP from 5.2 to 9.4%). Doubling irrigation rates did not double SAR and ESP in the soil (SAR 1.94 and 2.16 in 836- and 1672-mm/yr treatments in the Fluvial Recent soil, respectively, and ESP 12.5 and 13.4% in the same treatments). Even in the treatments with the highest rate of TME application, ESP remains lower than 15%, a sodicity risk level of “none to slight” (Abrol et al. 1988). In spite of this Na accumulation, only the topsoil of the Fluvial Recent soil increased the EC with higher TME application rates (Fig. S5). EC in Fragic Pallic soil and pH in both soil types were not affected by TME application. It is possible that changes would happen in the long term, as usually reported by other authors (Bedbabis et al. 2015; Mohammad Rusan et al. 2007; Qian and Mecham 2005).

Although Na accumulation in the soil did not perturb drainage in this experiment, over the long term, a reduction in infiltration rates may occur (Assouline and Narkis 2011; Bedbabis et al. 2014). In that case, the soils may require periodic amendments with gypsum or dolomite to maintain structure (Abrol et al. 1988).

Boron and trace elements are occasionally a concern for the reuse of TME (Angin et al. 2005; Pedrero et al. 2010). On some occasions, crop yield has been decreased by the application of TME due to toxicity of one or various elements, as reviewed by Pedrero et al. (2010) and reported by Chatzakis et al. (2011). In this experiment, the concentrations of B, Al, Cd, Cu, Fe, Mn, As and Zn in TME were below detection limits (< 0.001 mg/Cd/L) or much lower than the recommended limits (Table 1) for use in irrigation according to EPA (EPA 2004). Cd was not detected in the leachates or pasture (< 0.001 mg/kg), and B, Al, Cu, Fe, Mn and Zn concentrations were not higher in TME irrigated treatments than in controls.

Although they were not an objective of this experiment, further consideration of emerging organic contaminants would increase the understanding of potential risks of TME irrigation into food chains or receiving environments (González García et al. 2019; Hurtado et al. 2016; Martínez-Piernas et al. 2018).

## Conclusions and recommendations

The TME from Duvauchelle treatment plant was suitable for pasture irrigation in both the Fluvial Recent soil and the Fragic Pallic soil. This experiment demonstrated that irrigation was fundamental for keeping the pasture production during the summer months, which recommends TME as an alternative water source in climate change scenarios with reduced water availability. TME irrigation would decrease the need of mineral fertilizers, and at a rate of 800 mm/yr, it could save about US$ 840 ha/yr in fertilizing with N, P, S, K, Ca and Mg. In case of a constant irrigation rate during the year, up to 800 mm/yr would be an optimal option, with an increase in pasture production, compared with non-irrigation, by 65%, in the case of fescue/brown top over Fluvial Recent soil lysimeters, and by 121%, in case of ryegrass over the Fragic Pallic soil lysimeters. At this application rate, the amount of N leached (1 kg/N/ha) was insignificant compared with grazed pastures (~ 40 kg/N/ha). Adapting the irrigation rate in different seasons would allow to increase irrigation during summer up to 1500 mm/yr without risk of nutrient leaching and to increase pasture production to an extra of 57%.

It is unlikely that in the medium term (~ 20 years), P or Na accumulation in the soil will be a problem. In the worst case scenario of applying 75 kg/P/ha/yr, the total P concentration in the topsoil will rise to 57% over 50 years in the case of ryegrass over Fragic Pallic soil and 100% over 50 years in the case of fescue/browntop over Fluvial Recent soil. The final concentrations of the total P and Olsen P calculated for both scenarios were comparable with those on productive pastures in New Zealand. Although Na accumulated in the soil columns in all the TME treatments, the rate of accumulation was not proportional to the TME application rate, which indicates that Na was moving down through the soil profile and leaching. During the experiment, there was no evidence of ponding or decreased infiltration capacity. Na concentration in pasture increased with higher TME application rates. Highly productive cut and carry pasture, such as ryegrass for feed instead of fescue/browntop for turf, would be the best option for removing the N, P and Na supplied by TME. The amount of P, Ca, S, K, Mg and Na applied was higher than the uptake by pasture and the total leached in all the TME irrigated treatments. The application of any element to a system at a rate that is greater than the rate that is removed is ultimately unsustainable. The TME used in this experiment did not pose any risk related with trace elements. However, future work should investigate fluxes of contaminants associated with pharmaceuticals and/or personal care products in TME-irrigated soils.

## Electronic supplementary material

ESM 1 (PDF 1418 kb)
