# Supplementary material for: Risks and benefits of pasture irrigation using treated municipal effluent : a lysimeter case study, Canterbury, New Zealand
Source: Environ Sci Pollut Res Int. 2020 Jan 23;27(11):11830–41. doi: 10.1007/s11356-020-07759-8 (PMC7136187; doi:10.1007/s11356-020-07759-8)
Supplement: Supplementary file 1 — (PDF 1418 kb) [file 11356_2020_7759_MOESM1_ESM.pdf]

Supplementary Material

Risks and benefits of pasture irrigation using treated municipal effluent: a lysimeter case study, Canterbury, New Zealand

GUTIERREZ-GINES Maria Jesus, MISHRA Minakshi, MCINTYRE Cameron, CHAU Henry Wai, ESPERSCHUETZ Juergen, MCLENAGHEN Roger, BOURKE Mike P, ROBINSON Brett H

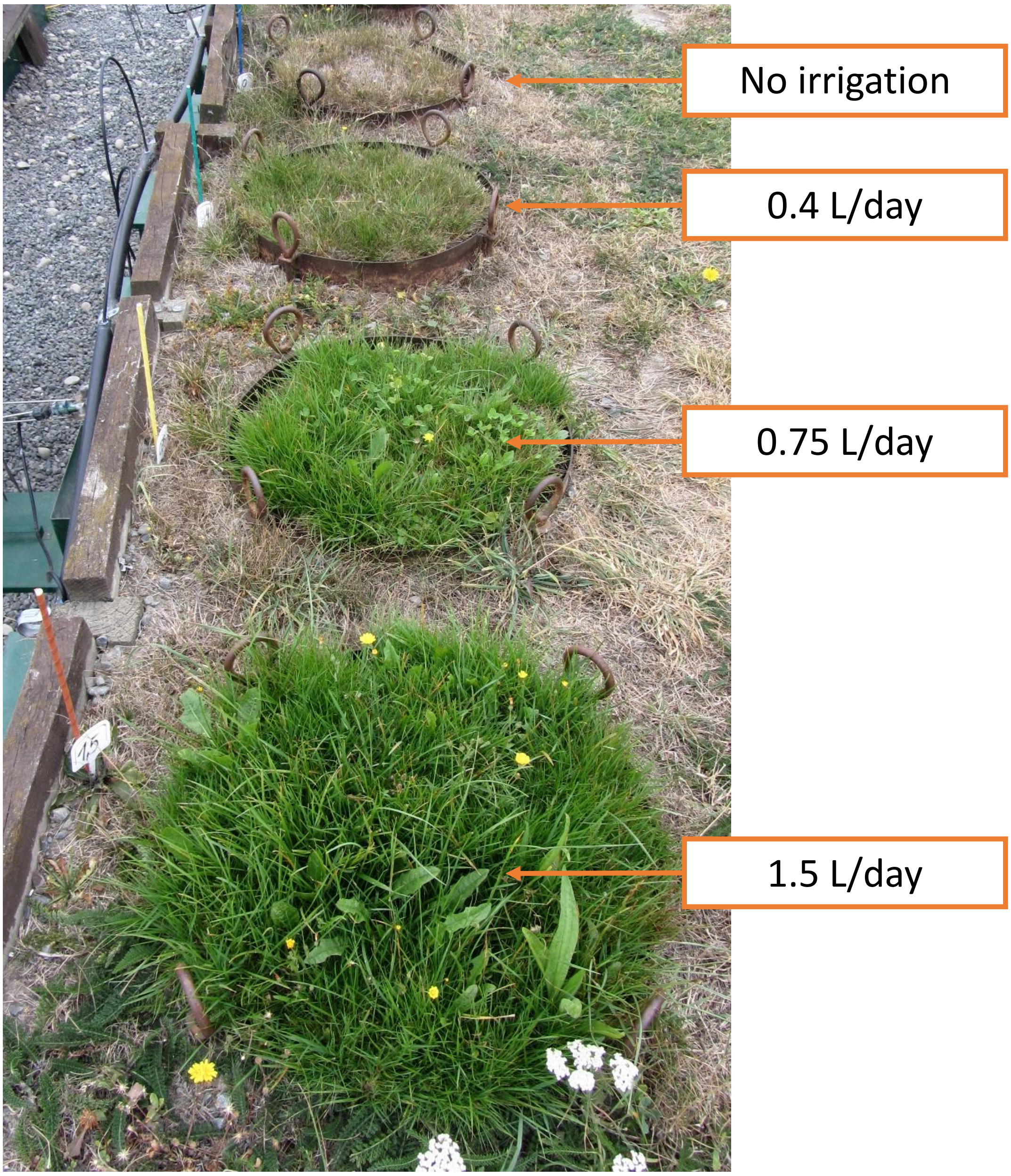

Fig. S1. Pasture growth on four lysimeters containing Fluvial Recent soil in February 2016. The figures to the right of the picture indicate the volume of treated effluent that the lysimeter was receiving daily Monday – Friday.

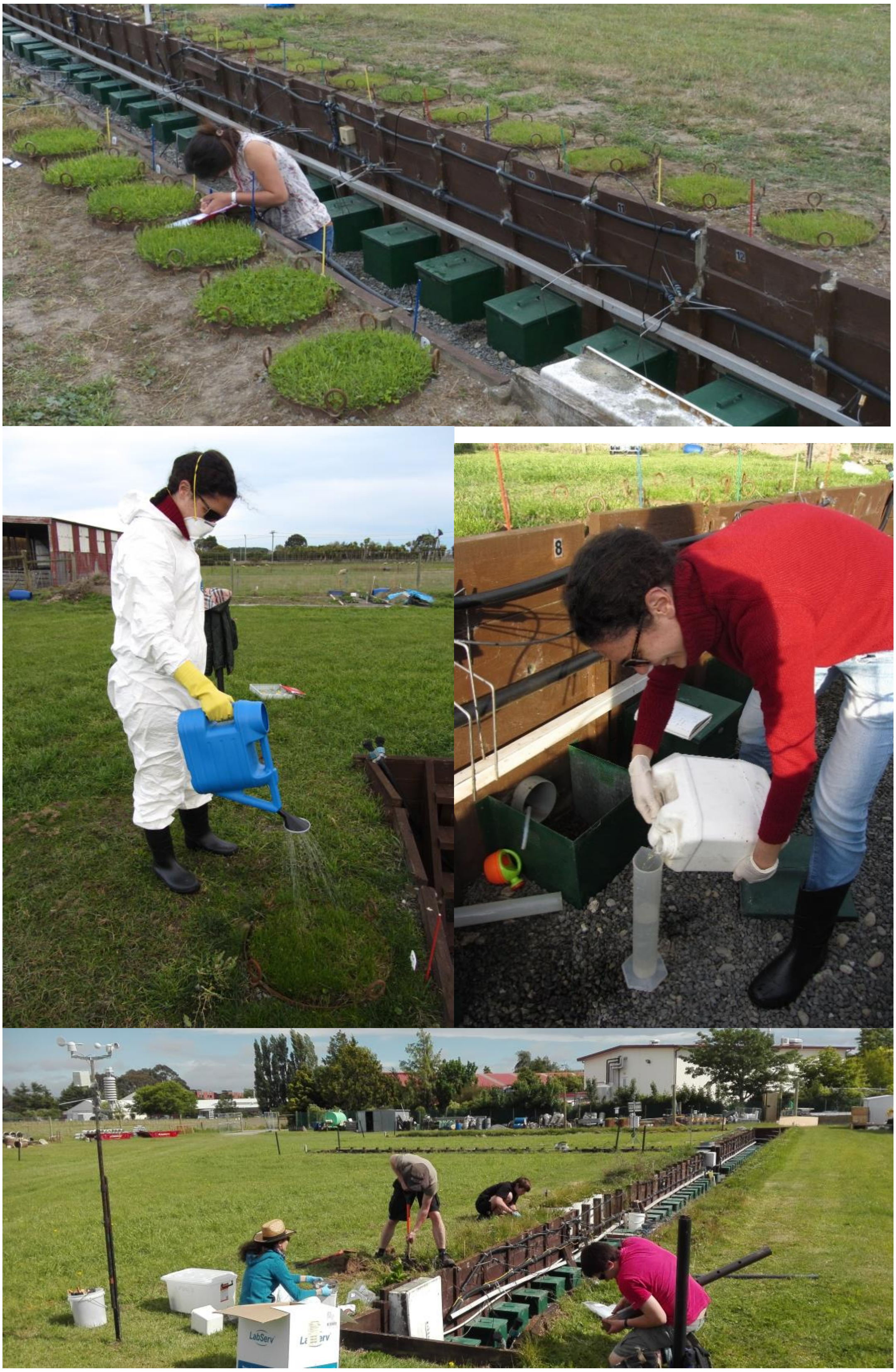

Fig. S2. Top: The installed lysimeters showing the six Fragic Pallic soil cores (front-left) and the 12 Fluvial Recent soil cores (rear-right). Centre left: Effluent application. Centre right: Drainage collection. Bottom: Destructive sampling of the lysimeters at the conclusion of the experiment. 16th of November, 2016.

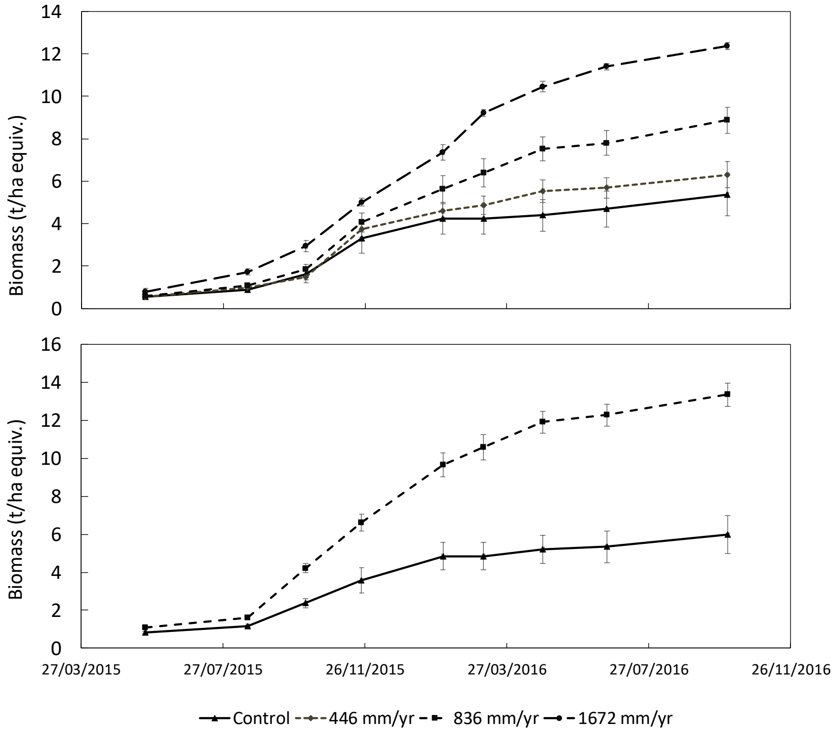

Fig. S3. Cumulative biomass production in the lysimeter experiment for the Fluvial Recent soil (top) and Fragic Pallic soil (bottom), expressed as tonnes per hectare equivalent. Bars represent the standard error of the mean (n=3).

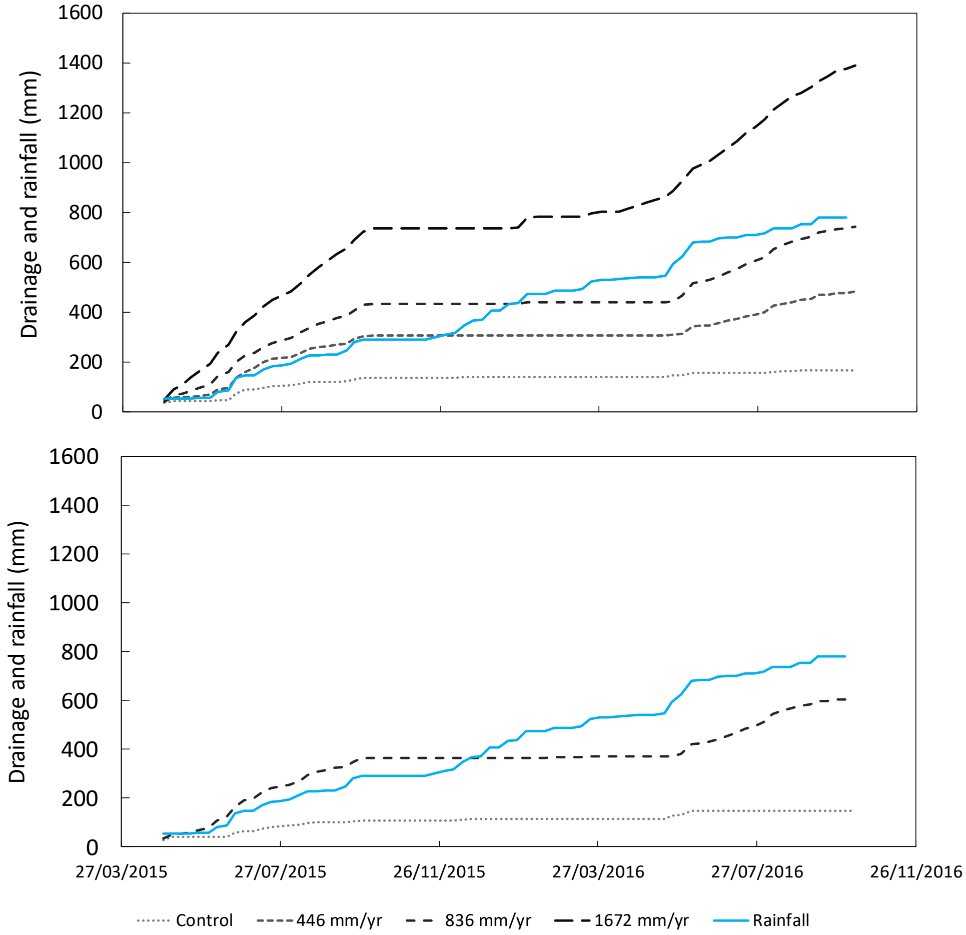

Fig. S4. Cumulative rainfall, and drainage from the lysimeters for the Fluvial Recent soil (top) and Fragic Pallic soil (bottom).

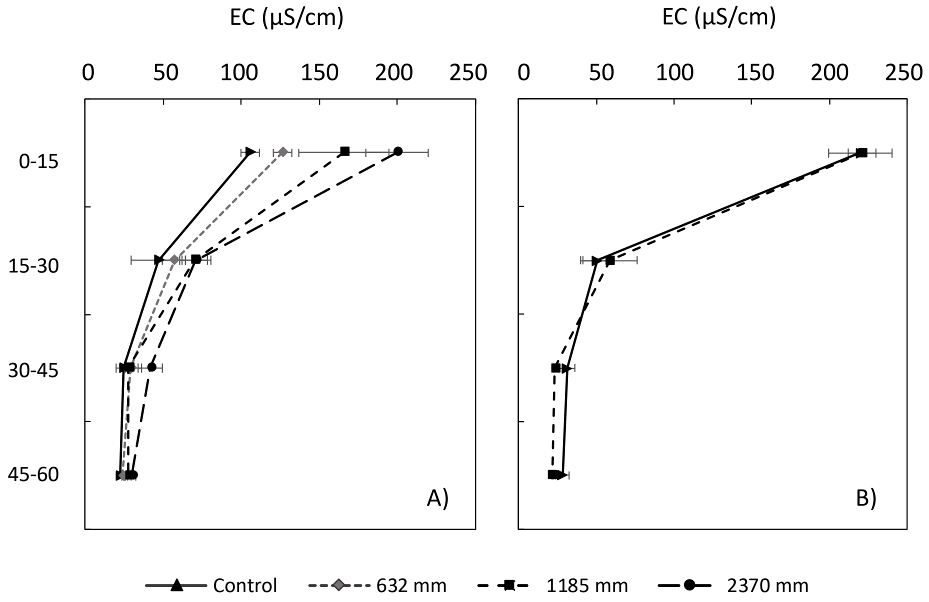

Fig. S5. Electrical conductivity as a function of depth at the end of lysimeter experiment for the Fluvial Recent soil (A) and Fragic Pallic soil (B). Bars represent the standard error of the mean (n=3).

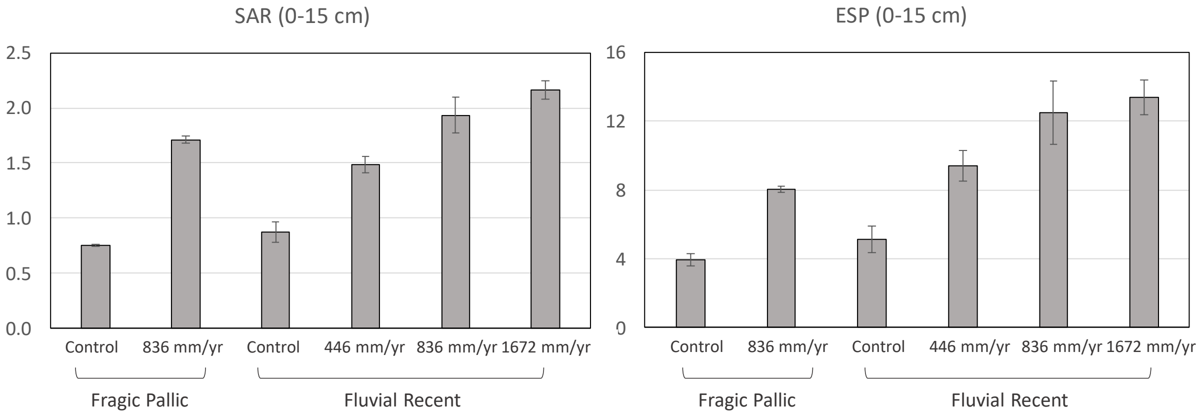

Fig. S6. Sodium Adsorption Ratio (SAR) and Exchangeable Sodium Percentage (ESP) in the topsoil of the lysimeters after 18 months of TMW irrigation. Bars represent average and error bars, standard error.

Table S1. Mass of sulphur (kg/ha equiv) in the treated municipal wastewater, pasture, soil and drainage water over the entire lysimeter experiment. Values in brackets represent the standard error of the mean (n=3). For each soil type, values with the same letter are not significantly different. The Fluvial Recent soil and Fragic Pallic soil were tested independently.

	Irrigation S (kg/ha equiv.)	Pasture S (mg/kg)	Pasture S (kg/ha equiv.)	S leached (kg/ha equiv.)	Soil S (0 – 60 cm) (kg/ha equiv.)
Fluvial Recent soil					
Control	0	2376 (40)^{a}	14 (3)^{a}	7 (2)^{a}	2389 (169)^{a}
446 mm/yr	169	2653 (169)^{a}	17 (2)^{ab}	21 (5)^{ab}	2190 (168)^{a}
836 mm/yr	317	2649 (113)^{a}	24 (2)^{b}	40 (11)^{ab}	2065 (75)^{a}
1672 mm/yr	634	2676 (60)^{a}	35 (2)^{c}	67 (12)^{b}	2294 (124)^{a}
Fragic Pallic soil					
Control	0	2941 (164)^{a}	17 (2)^{a}	11 (1)^{a}	2275 (96)^{a}
836 mm/yr	317	3111 (76)^{a}	40 (0)^{b}	45 (8)^{b}	1989 (196)^{a}

Table S2. Mass of potassium (kg/ha equiv) in the treated municipal wastewater, pasture, soil and drainage water over the entire lysimeter experiment. Values in brackets represent the standard error of the mean (n=3). For each soil type, values with the same letter are not significantly different. The Fluvial Recent soil and Fragic Pallic soil were tested independently.

	Irrigation K (kg/ha equiv.)	Pasture K (mg/kg)	Pasture K (kg/ha equiv.)	K leached (kg/ha equiv.)	Soil K (0 – 60 cm) (kg/ha equiv.)
Fluvial Recent soil					
Control	0	12690 (917)[a]	65 (12)[a]	1 (0.3)[a]	34597 (493)[a]
446 mm/yr	177	12270 (714)[a]	68 (4)[a]	2 (0.3)[a]	34848 (785)[a]
836 mm/yr	331	13450 (602)[a]	112 (8)[b]	3 (0.4)[a]	35627 (908)[a]
1672 mm/yr	662	14860 (541)[a]	179 (6)[c]	4 (0.1)[a]	35165 (1134)[a]
Fragic Pallic soil					
Control	0	16850 (995)[a]	104 (15)[a]	6 (2)[a]	40824 (1322)[a]
836 mm/yr	331	16630 (558)[a]	229 (16)[b]	21 (6)[a]	37392 (3319)[a]

Table S3. Mass of calcium (kg/ha equiv) in the treated municipal wastewater, pasture, soil and drainage water over the entire lysimeter experiment. Values in brackets represent the standard error of the mean (n=3). For each soil type, values with the same letter are not significantly different. The Fluvial Recent soil and Fragic Pallic soil were tested independently.

	Irrigation Ca (kg/ha equiv.)	Pasture Ca (mg/kg)	Pasture Ca (kg/ha equiv.)	Ca leached (kg/ha equiv.)	Soil Ca (0 – 60 cm) (kg/ha equiv.)
Fluvial Recent soil					
Control	0	3879 (527)[a]	24 (5)[a]	19 (5)[a]	48351 (1620)[a]
446 mm/yr	371	3373 (216)[a]	26 (4)[a]	51 (12)[ab]	46775 (748)[a]
836 mm/yr	696	3350 (69)[a]	39 (3)[ab]	56 (9)[bc]	47506 (1059)[a]
1672 mm/yr	1392	3327 (170)[a]	51 (0)[b]	95 (16)[c]	48786 (1433)[a]
Fragic Pallic soil					
Control	0	5581 (396)[a]	31 (2)[a]	19 (6)[a]	53218 (3475)[a]
836 mm/yr	696	4890 (183)[a]	68 (2)[b]	84 (1)[b]	49948 (4004)[a]

Table S4. Mass of magnesium (kg/ha equiv) in the treated municipal wastewater, pasture, soil and drainage water over the entire lysimeter experiment. Values in brackets represent the standard error of the mean (n=3). For each soil type, values with the same letter are not significantly different. The Fluvial Recent soil and Fragic Pallic soil were tested independently.

	Irrigation Mg (kg/ha equiv.)	Pasture Mg (mg/kg)	Pasture Mg (kg/ha equiv.)	Mg leached (kg/ha equiv.)	Soil Mg (0 – 60 cm) (kg/ha equiv.)
Fluvial Recent soil					
Control	0	2065 (279)[a]	13 (3)[a]	6 (1)[a]	33017[a]
446 mm/yr	124	1823 (110)[a]	15 (2)[a]	21 (7)[a]	32580[a]
836 mm/yr	232	1964 (52)[a]	23 (1)[ab]	23 (1)[a]	32074[a]
1672 mm/yr	463	1960 (210)[a]	33 (3)[b]	50 (17)[a]	32469[a]
Fragic Pallic soil					
Control	0	2481 (106)[a]	16 (1)[a]	5 (1)[a]	42274 (2734)[a]
836 mm/yr	232	2572 (78)[a]	38 (2)[b]	30 (2)[a]	40351 (2596)[a]
